# Epidemiology and clinical features of Skin and Soft Tissue Infections Caused by PVL-Positive and PVL-Negative Methicillin-Resistant *Staphylococcus aureus* Isolates in inpatients in China: a single-center retrospective 7-year study

**DOI:** 10.1080/22221751.2024.2316809

**Published:** 2024-02-07

**Authors:** Ye Jin, Wangxiao Zhou, Qi Ge, Ping Shen, Yonghong Xiao

**Affiliations:** a Department of General Intensive Care Unit, the Second Affiliated Hospital of Zhejiang University School of Medicine, Hangzhou, Zhejiang, People’s Republic of China; b State Key Laboratory for Diagnosis and Treatment of Infectious Diseases, National Clinical Research Center for Infectious Diseases, Collaborative Innovation Center for Diagnosis and Treatment of Infectious Diseases, the First Affiliated Hospital, Zhejiang University School of Medicine, Hangzhou, People’s Republic of China; c Key Laboratory of Early Warning and Intervention of Multiple Organ Failure, China National Ministry of Education, Hangzhou, Zhejiang, People’s Republic of China

**Keywords:** Skin and soft tissue infections, Panton-Valentine leucocidin, MRSA, Phage type, ST59

## Abstract

Previous studies have mainly focused on outpatient cases of skin and soft tissue infections (SSTIs), with limited attention to inpatient occurrences. Thus, we aimed to compare the clinical parameters of inpatients with SSTIs, performed genomic characterization, and determined the subtypes of Panton-Valentine leucocidin (PVL) bacteriophages of methicillin-resistant *Staphylococcus aureus* (MRSA) strains isolated from these patients. We found that PVL-positive patients had shorter hospital stays (mean, 9 vs. 24 days; *p* < 0.001) and abscess resolution durations (mean, 8 vs. 13 days; *p* < 0.01). PVL-positive MRSA-induced SSTIs were more frequently associated with abscesses [36/55 (65.5%) vs. 15/124 (12.1%), *p* < 0.001], with 52.7% undergoing incision and drainage; over 80% of PVL-negative patients received incision, drainage, and antibiotics. In PVL-positive patients receiving empirical antibiotics, anti-staphylococcal agents such as vancomycin and linezolid were administered less frequently (32.7%, 18/55) than in PVL-negative patients (74.2%, 92/124), indicating that patients with PVL-positive SSTIs are more likely to require surgical drainage rather than antimicrobial treatment. We also found that the ST59 lineage was predominant, regardless of PVL status (41.3%, 74/179). Additionally, we investigated the linear structure of the *lukSF-PV* gene, revealing that major clusters were associated with specific STs, suggesting independent acquisition of PVL by different strain types and indicating that significant diversity was observed even within PVL-positive strains detected in the same facility. Overall, our study provides comprehensive insights into the clinical, genetic, and phage-related aspects of MRSA-induced SSTIs in hospitalized patients and contributes to a more profound understanding of the epidemiology and evolution of these pathogens in the Chinese population.

## Introduction

*Staphylococcus aureus* colonizes the skin and mucosal surfaces in approximately 30% of healthy individuals [1] and is the primary aetiological agent of mild to moderate skin and soft tissue infections (SSTIs). Several virulence factors, including α-haemolysin, exfoliative toxins, Panton–Valentine leucocidin (PVL), or phenol-soluble modulins, have been linked to the severity of SSTIs [2–5].

PVL comprises two subunits: LukS-PV and LukF-PV. The genetic code for PVL involves two cotranscribed open reading frames, *lukS* and *lukF*, located on a lysogenic bacteriophage that can integrate into specific sites on the *S. aureus* chromosome [6]. Upon integration, these subunits collectively assemble into biologically active heptamers, inducing the lysis of human leukocytes, including granulocytes, monocytes, and macrophages, via pore formation and membrane damage [7]. The resulting leukocyte apoptosis leads to the release of inflammatory mediators, such as reactive oxygen species and cytokines, causing damage to epithelial cells and surrounding tissues [8]. Notably, PVL formation is independent of methicillin resistance. Both methicillin-resistant (MRSA) and methicillin-sensitive (MSSA) *S. aureus* strains produce PVL [9].

PVL detection has substantial clinical and therapeutic implications. Clinical and epidemiological evidence indicates that PVL has profound implications for deep-seated abscesses, multifocal lesions, recurrent SSTIs, resistance to multiple antibiotics, and SSTI outbreaks [10–12]. SSTIs represent a substantial proportion of primary care consultations, with most studies focusing primarily on outpatients [13]. Although studies involving PVL often use MRSA as an inclusion criterion [14], there is a noticeable lack of data comparing the epidemiology and clinical features of SSTIs caused by PVL-positive and PVL-negative MRSA isolates among inpatients.

This study aimed to compare the clinical characteristics of SSTIs caused by PVL-positive and PVL-negative MRSA isolates among hospitalized patients, compare the genomic epidemiology and characteristics of PVL-positive and PVL-negative MRSA strains, and subtype PVL bacteriophages carried by MRSA isolates from hospitalized patients. Our study provides comprehensive insights into the clinical, genetic, and phage-related aspects of MRSA-induced SSTIs in hospitalized patients, contributing to a more profound understanding of the epidemiology and evolution of these pathogens in the Chinese population.

## Materials and methods

### Study design

This study investigated inpatients diagnosed with SSTIs at the First Affiliated Hospital of Zhejiang University School of Medicine, a 5,500-bed public teaching hospital located in east China that receives approximately 10 million patients per year. We retrospectively analyzed the data extracted from the hospital’s electronic medical record system covering the period from January 2014 to December 2020. The use of a large, representative sample from a single, well-established hospital ensured the generalisability of the findings to a wider population.

This study has obtained ethical approval from the Ethics Committee of the First Affiliated Hospital of Zhejiang University School of Medicine, under file batch number IIT20210311B.

### Study definitions

Patients who met the following four criteria were eligible for inclusion in the study:

- Patients diagnosed with SSTIs, including cellulitis, skin abscesses, infected skin ulcers, infected surgical incisions, infected traumatic wounds, diabetic foot ulcers, decubitus ulcers, and ischaemic ulcers. Patients were excluded if they presented with uncomplicated skin or superficial skin structural infections, such as minor skin abrasions.
- Patients whose SSTIs were caused by MRSA.
- MRSA must have been isolated from inpatient specimens only.
- Genetic analysis must have been performed for the isolated MRSA strains.

These criteria were established to ensure that the study focused on a homogeneous patient population with clear indications of MRSA infection and to facilitate an accurate and reliable analysis of the isolated MRSA strains.

### Antimicrobial susceptibility determination

Identification and antimicrobial susceptibility testing of the bacterial isolates were conducted using the VITEK 2 system (bioMérieux, Marcy-l’ Etoile, France) following the guidelines provided by the Clinical and Laboratory Standards Institute.

#### Community-acquired (CA)-MRSA definition

CA-MRSA was defined as a strain isolated within the initial 48 h from outpatients or hospital-admitted patients, specifically those lacking any record of inpatient contact with nursing homes, shelters, or other healthcare facilities in the preceding 12 months. Furthermore, these individuals should not possess indwelling catheters or artificial medical devices.

### Whole-genome sequencing and molecular analysis of MRSA strains

Genomic DNA from 179 PVL-positive and PVL-negative MRSA isolates was extracted using Gentra Puregene Yeast/Bact. software (Qiagen, San Francisco/Bay Area, CA, USA). A sequencing library was prepared using a Nextera XT kit (Illumina, San Diego, CA, USA). Genomes were sequenced using an Illumina Hiseq X-ten system (Illumina) with 2 × 150-bp paired-end libraries. SPAdes v3.14.1 [15] was used to assemble the clean reads obtained after adaptor trimming and quality filtering using fastp v0.20.1 [16] with default parameters (except Phred quality score ≥ 20). MLST v2.0 (https://cge.food.dtu.dk/services/MLST/), SpaFinder v1.0 (https://cge.food.dtu.dk/services/spaTyper/), and SCCmecFinder v1.2 (https://cge.food.dtu.dk/services/SCCmecFinder/) were used to confirm the sequence types, *spa* types, and SCC*mec* types of MRSA isolates with default parameters, respectively. MRSA isolates were assigned to clonal complexes (CCs) when they share six of the seven alleles (based on multilocus sequence typing) with the founder sequence type using PHYLOViZ v2.0, with the geoBURST Full MST algorithm [17]. Antimicrobial resistance genes and chromosomal point mutations carried by the strains were analyzed using the web-based ResFinder v4.4.2 [18] (http://genepi.food.dtu.dk/resfinder), with a threshold setting of 90% sequence similarity and 80% sequence coverage. Virulence factors were identified using ABRicate v1.0.0 (https://github.com/tseemann/abricate) with the VFDB database (80% identity and 80% query coverage cutoffs) [19]. The segments of the PVL-encoding prophages in each genome were successfully assembled using the assembly graph viewer Bandage v0.9.0 [20]. By displaying connections that were not present in the contig file (produced using SPAdes v3.14.1), we manually extracted candidate prophage-related sequences and successfully completed all *de novo* assemblies for PVL-encoding prophages.

### Phylogenetic analysis of MRSA isolates

Panaroo v1.1.3 [21] was used to extract the core genome from all 179 MRSA isolates, followed by extracting single-nucleotide polymorphism (SNP) alignments from the core genome alignment using snp-sites v2.5.1 [22]. A maximum likelihood phylogenetic tree was constructed using RAxML v8.2.11 [23] employing a GTR model with 1,000 bootstrap replications. Additionally, a minimum spanning tree based on SNP data for all 135 hospital-acquired MRSA (HA-MRSA) isolates was reconstructed using PHYLOViZ v2.0, with the geoBURST Full MST algorithm [17].

### Nucleotide sequence accession numbers

The whole-genome sequences of isolates determined in this study were submitted to the DDBJ/EMBL/GenBank database under the BioProject accession no. PRJNA1054793.

### Statistical analysis

Logistic regression analysis was used to calculate the odds ratios (ORs), 95% confidence intervals (CIs), and *p*-values for both case-control comparisons and assessments of clinical characteristics based on PVL status. Categorical variables, including age groups, were compared using the χ^2^ or Fisher exact test. The predetermined significance level was set at *p* < 0.05. Additionally, Wilcoxon rank-sum tests were performed to compare antimicrobial resistance and virulence genes between groups.

## Results

### Epidemiological and clinical features of SSTIs caused by PVL-positive and PVL-negative MRSA

**Figure 1.**
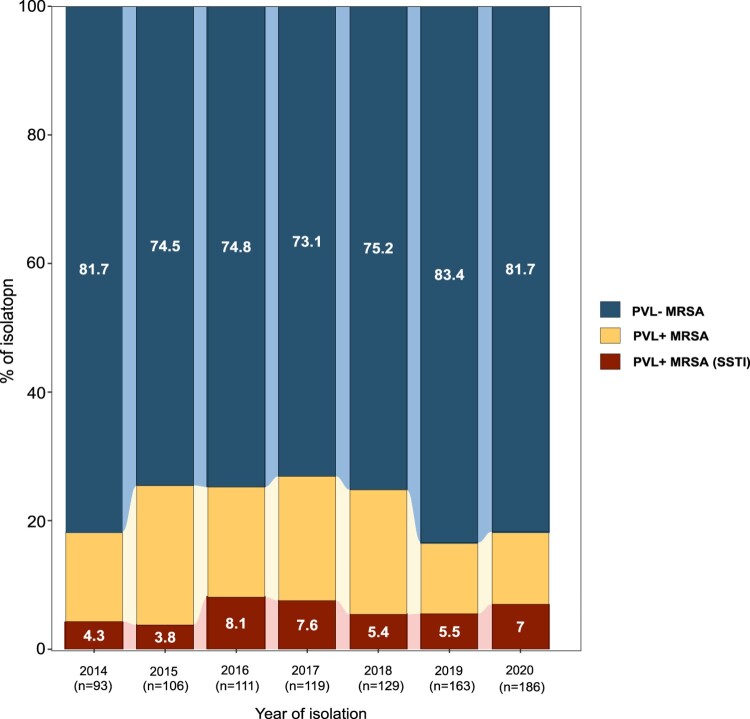
Annual proportions of PVL-positive and PVL-negative methicillin-resistant *Staphylococcus aureus* (MRSA) from 2014 to 2020.

A total of 907 non-repetitive MRSA strains (excluding those isolated from sputum) were isolated between 2014 and 2020, of which 197 (21.7%) carried the *pvl* gene (Figure 1). Notably, 179 cases of SSTIs caused by MRSA were identified over 7 years, fifty-five (30.7%) MRSA isolates carried the *pvl* gene. There were no significant changes in the proportions of PVL-positive and PVL-negative MRSA from 2014 to 2020 (Figure 1). Table 1 presents the characteristics of patients with MRSA-induced SSTIs. Notably, patients diagnosed with SSTIs attributed to PVL-positive MRSA were younger than their PVL-negative counterparts. The average age of patients diagnosed with MRSA-induced SSTIs was 47 years (range, 1–78 years). Moreover, the incidence of PVL-positive MRSA-induced SSTIs was the highest in subjects aged 18–40 years, decreasing with advancing age (OR, 2.3; 95% CI, 1.1–5.7; *p* = 0.03, test for trend). In contrast, individuals with PVL-negative SSTIs tended to be older, with 41.1% of cases occurring among individuals aged >60 years.

**Table 1.** Characteristics of PVL-positive and PVL-negative MRSA from SSTIs from inpatient.

Parameter/Category	PVL-positive	PVL-negative	*p*-value
	(*n* = 55)	(*n* = 124)	
Male sex, n (%)	23 (41.8)	63 (50.8)	0.47
Age, mean years (range)	46 (1-78)	62 (1-82)	0.008
Age 0–18 years	6 (10.9)	6 (4.8)	0.042
>18–40 years	25 (45.5)	30 (24.2)	0.035
>40–60 years	16 (29.1)	51 (41.1)	0.046
>60 years	8 (14.5)	37 (29.8)	0.023
Community-acquired	33 (60.0)	11 (8.9)	0.0001
AIDS	0	1	NA
Diabetes mellitus	6	30	NA
Chronic obstructive pulmonary diseases	0	5	NA
Connective tissue diseases	0	1	NA
End-stage renal disease	0	1	NA
End-stage kidney disease	3	18	NA
Hematological diseases	0	24	NA
Cancer	2	18	NA
Surgical history	3	38	NA
Primary infection	38 (69.1)	12 (21.8)	0.002
Diabetic foot ulcer	6 (10.9)	30 (24.2)	0.038
Ischemic ulcer	0	9 (7.3)	0.001
Surgical site infections	3 (5.5)	38 (30.6)	0.001
Infected traumatic wound	8 (14.5)	28 (22.6)	0.042
Skin abscesses	17 (30.9)	12 (9.7)	0.001
Mastitis	6 (10.9)	1 (0.8)	0.001
Furuncles	9 (16.4)	0	0.001
Others	8 (14.5)	5 (4.0)	NA

Note: NA, not applicable.

Comparatively, a significantly larger proportion of PVL-positive MRSA infections originated in the community than infections caused by PVL-negative MRSA strains (60.0% vs. 8.9%; *p* < 0.001). Additionally, PVL-negative MRSA isolates that caused SSTIs were primarily associated with patients with a history of surgery, chronic renal disease, diabetes, cancer, or immunosuppression, whereas PVL-positive MRSA isolates tended to infect previously healthy individuals (Table 1). PVL-positive MRSA were isolated from eight ward areas, but were mainly from the orthopaedics, hand surgery, and dermatology departments. The types of SSTIs significantly differed between the two populations (*p* < 0.001). Patients infected with PVL-negative MRSA had a higher prevalence of diabetic foot (24.2%), infected traumatic wounds (22.6%), and surgical site infections (30.6%). In contrast, patients with PVL-positive MRSA infections exhibited a higher incidence of skin abscess (30.9%), furuncles (16.4%), mastitis (10.9%), and other severe localized pathology, suggesting that PVL-positive infections were more frequently associated with abscesses [36/55 (65.5%) vs. 15/124 (12.1%), *p* < 0.001].

In contrast to individuals infected with PVL-negative MRSA, those harbouring PVL-positive MRSA demonstrated a reduced hospitalization duration (mean, 9 days vs. 24 days; *p* < 0.001) and a shorter abscess resolution period (mean, 8 days vs. 13 days; *p* < 0.01) (Table 2). However, these assessments were not adjusted for potential confounding variables such as age.

**Table 2.** Comparison of therapy between PVL-positive SSTIs and PVL-negative SSTIs.

Parameter/Category	PVL-positive	PVL-negative	*p*-value
	(*n* = 55)	(*n* = 124)	
Duration of hospitalization (days), mean (range)	9 (4-26)	24 (6-128)	<0.001
Duration of SSTIs (days), mean (range)	8 (3-19)	13 (6-39)	<0.05
Surgical drainage alone, n (%)	29 (52.7)	10 (8.1)	<0.001
Vancomycin or linezolid n (%)	18 (32.7)	92 (74.2)	<0.001
Antimicrobial therapy alone, n (%)	6 (10.9)	26 (21.0)	<0.05
Vancomycin or linezolid alone	2 (3.6)	8 (6.5)	<0.05
Combined with another one antibiotic	2 (7.3)	12 (9.7)	<0.001
Combined with another two antibiotics	0	3 (2.4)	<0.05
Combined with another four antibiotics	0	0	NA
Other antibiotics alone	2 (3.6)	3 (2.4)	>0.05
Surgical drainage combined with antimicrobial therapy, n (%)	20 (36.4)	101 (81.5)	<0.05
Combined with another antibiotics	6 (10.9)	32 (25.8)	>0.05
Combined with vancomycin or linezolid	14 (25.5)	69 (55.6)	<0.05

Note: NA, not applicable.

Treatment strategies varied among the 55 patients diagnosed with PVL-positive MRSA-induced SSTIs. Specifically, 49 (89.1%) underwent incision and drainage in conjunction with antibiotic therapy, 6 (10.9%) received antibiotics as the sole treatment, and 29 (52.7%) exclusively received incision and drainage. However, a substantial proportion of PVL-negative patients (80.0%) underwent a combined approach involving incision and drainage procedures along with antibiotic therapy, often employing broad-spectrum antibiotics. Vancomycin and levofloxacin are the most frequently used agents for SSTI therapy. Interestingly, among the PVL-positive patients who received empirical antibiotics, few were treated with anti-staphylococcal agents, such as vancomycin and linezolid. Specifically, only 18/55 (32.7%) PVL-positive patients were treated with these agents, compared to 92/124 (74.2%) PVL-negative patients. Of the PVL-positive cases, 38 (69.1%) were classified as primary infections, and 8 (14.5%) were classified as deep secondary infections. In contrast, more than half of the SSTIs caused by PVL-negative MRSA strains were attributed to secondary infections, such as surgical and trauma site infections.

### Characterization of the PVL-positive and PVL-negative MRSA isolates

**Figure 2.**
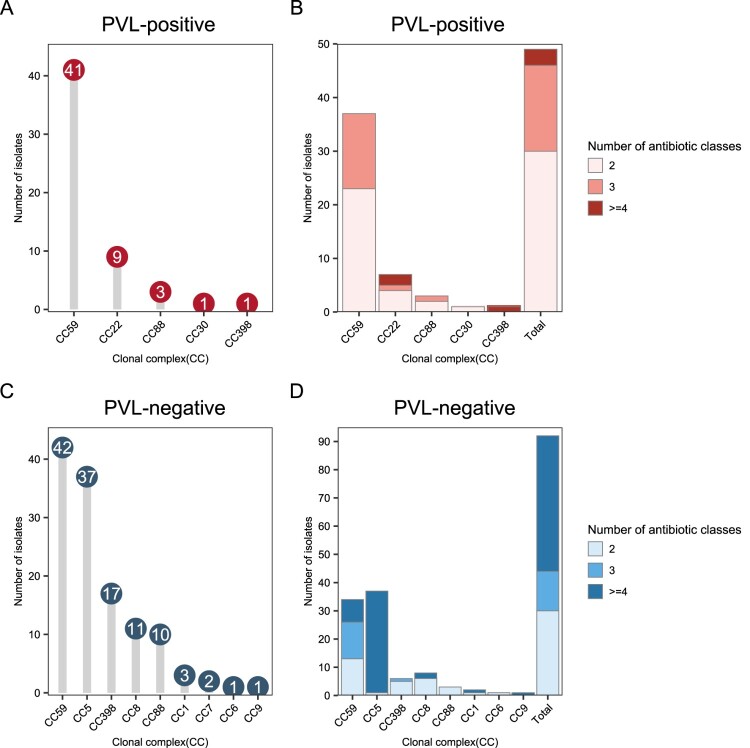
Numbers and resistance profiles of methicillin-resistant *Staphylococcus aureus* (MRSA) isolates across different clonal complexes (CCs) between the PVL-positive and PVL-negative strains. (A) Numbers of MRSA strains identified for each CC across the PVL-positive lineage. (B) Distribution of resistance to multiple antibiotic classes across different CCs in the PVL-positive lineage. (C) Numbers of MRSA strains identified for each CC across the PVL-negative lineage. (D) Distribution of resistance to multiple antibiotic classes across different CCs in the PVL- negative lineage.

**Figure 3.**
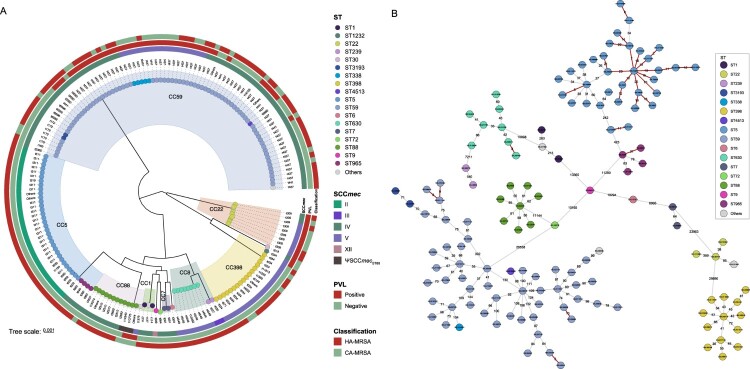
Characterization of 179 methicillin-resistant *Staphylococcus aureus* (MRSA) strains isolated from SSTIs. (A) Clonal complexes are marked using different background colour fills. The *SCCmec* type and presence of PVL are mapped on the tree (from inner to outer circle). Tips are coloured according to STs of strains. (B) A minimum spanning tree was constructed using 155 hospital-acquired MRSA isolates from our dataset. Each solid circle corresponds to one isolate, and the connecting lines display the number of single-nucleotide polymorphisms (SNPs) in pairwise comparisons. Red markings indicate potential transmission clusters, defined by a threshold of 24 SNPs.

Five CCs were identified among the PVL-positive isolates compared to nine CCs found among the PVL-negative strains. Notably, CC59 was the predominant clone detected in nearly half (46.4%, 83/179) of all MRSA isolates in our study, irrespective of their PVL status (Figure 2). Among the PVL-positive strains, CC59 (74.55%, 41/55) was the most prevalent MRSA lineage, followed by CC22 (9/55, 16.36%). In the PVL-negative samples, CC5 was the second most common CC, with an occurrence rate of 29.8% (37/124). Specifically, 47.3% of the PVL-positive MRSA strains harboured SCC*mec* type V, in contrast to the PVL-negative strains, of which only 21% harboured SCC*mec* type V (*p* < 0.05; Figure 3). Additionally, we observed that regardless of the PVL status, CA-MRSA exclusively carried SCC*mec* types V (*n* = 23, 52.3%) and IV (*n* = 18, 40.9%), along with the previously reported pseudo-SCC*mec*, referred to as ΨSCC*mec* ST88 (*n* = 3, 6.8%) [24].

To explore the transmission dynamics of hospital-acquired (HA)-MRSA strains, we performed a genomic analysis of 135 isolates obtained from the SSTI samples. Among the HA-MRSA clones, we quantified the number of SNPs in the isolated strains to identify potential transmission pairs and conducted an epidemiological investigation. Figure 3(B) illustrates the SNP distribution among the isolates of these clones, which ranged from 0 to 29,890 SNPs. Notably, the red line in the ST5 clone indicates SNPs < 24 [25], signifying a close genetic relationship among these strains and suggesting a strong possibility of nosocomial transmission.

All MRSA isolates were susceptible to vancomycin, linezolid, nitrofurantoin, quinupristin/dalfopristin, tigecycline, and daptomycin. Almost all were susceptible to rifampin (97.21%, 174/179), trimethoprim-sulphamethoxazole (94.97%, 170/179), and teicoplanin (99.44%, 178/179). Among PVL-positive MRSA strains, approximately 60% (33/55) exhibited susceptibility to non-β-lactamase agents (except erythromycin and clindamycin). In contrast, over 80% of the PVL-negative strains were resistant to at least one second-line agent, such as ciprofloxacin, levofloxacin, tetracycline, erythromycin, or clindamycin. Furthermore, over 70% (74.2%, 92/124) of the PVL-negative strains displayed resistance to two or more of these alternative oral antibiotics, and approximately half were resistant to three or more. Conversely, more than half (30/55, 54.5%) of the PVL-positive strains were resistant to only one non-β-lactamase agent. A few PVL-positive strains exhibited resistance to fluoroquinolones (3.6%, *n* = 2), whereas significantly more PVL-negative strains were resistant (37.9%, *n* = 47, *p* < 0.01). Among the MRSA strains isolated, only one exhibited resistance to mupirocin (minimum inhibitory concentration >512 mg/L) and belonged to CC8 (ST630). Eight strains belonging to CC8 (ST630) were resistant to fusidic acid.

Moreover, distinct STs are potentially correlated with specific categories of SSTIs. Patients infected with ST88 isolates primarily presented with otitis, pyomyositis, and cellulitis, whereas MRSA ST5 and PVL-negative ST59 strains were prevalent in postsurgical infections, posttraumatic wound infections, and patients with diabetes. ST59, ST398, and ST22 isolates were primarily associated with furuncles and mastitis, whereas other PVL-positive isolates were obtained from patients diagnosed with various conditions, including traumatic head or orthopaedic injury, postsurgical wounds, and other skin infections.

### Patterns of resistance and virulence genes vary between PVL-positive and PVL-negative MRSA isolates

**Figure 4.**
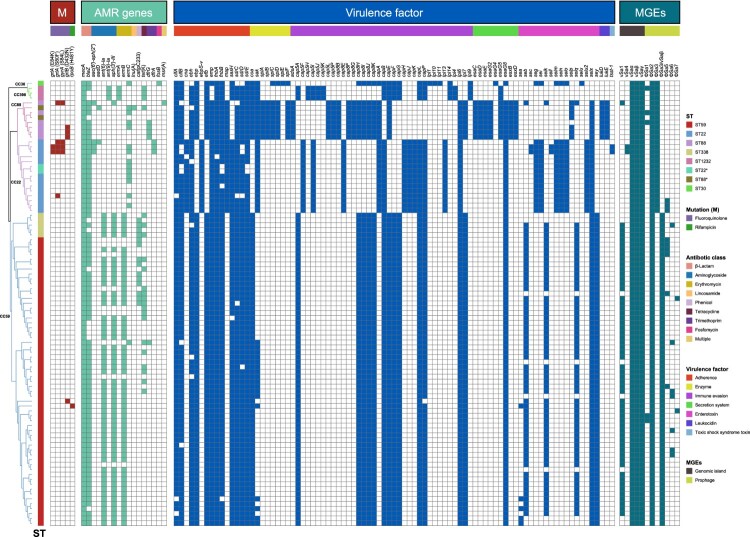
Distributions of resistance genes and virulence factors between PVL-positive and PVL-negative methicillin-resistant *Staphylococcus aureus* (MRSA) strains.

As shown in Figure 4, there was no significant difference in the number of resistance genes between PVL-positive and PVL-negative strains. In total, 22 resistance genes associated with macrolides (*ermA*, *ermB*, *ermC*, *ermT*), lincosamides (*lnu[A]*, *lnu[B]*, *lnu[G]*), aminoglycosides (*aac*[6′]*-aph*[2″], *aadD*, *aph(3′)-III*, *ant(6)-Ia*, *ant(9)-Ia*, *aph(2″)-Ia*), phenicol (*cat*), trimethoprim (*drfG*), tetracycline (*tetK*, *tetL*, *tetM*), antiseptic(*qacA*), mupirocin (*mupA*), fosfomycin (*fosB*, *fosD*), and fusidic acid (*fusB*) were detected in both PVL-positive and PVL-negative strains (Figure 4). Consistent with the results of the resistance genotypes, *fusB* was present in eight fusidic acid-resistant CC8 (ST630) clones, whereas the antibiotic resistance gene *qacA* was only detected in CC5 (ST5, *n* = 16; ST965, *n* = 1) and CC8 (ST630, *n* = 1) clones, which were all PVL negative.

Exfoliative toxin genes, such as *eta* and *etb,* were not detected in the analyzed strains. Regarding staphylococcal enterotoxin genes, 36.3% (45/124) of the PVL-negative strains carried *sea*, whereas 25.8% carried *seb* and *sec*. Among the PVL-positive strains, the majority lacked *sea* (*n* = 52, 94.5%) and *sec* (*n* = 54, 98.2%); however, 65.5% (36/55) contained *seb*. Additionally, enterotoxin genes exhibited high variability among MRSA isolates and were often associated with specific clonal lineages. The predominant toxin gene profile was *seb*-*selk*-*selq* in most STs of CC59, *sea-selk-selq* in most ST239, and *sec-sell* in ST5. The toxic shock syndrome toxin-encoding gene *tsst-1* was identified in most PVL-negative ST5 isolates (*n* = 34, 27.4%).

### Linear comparison of PVL prophages among PVL-positive strains

**Figure 5.**
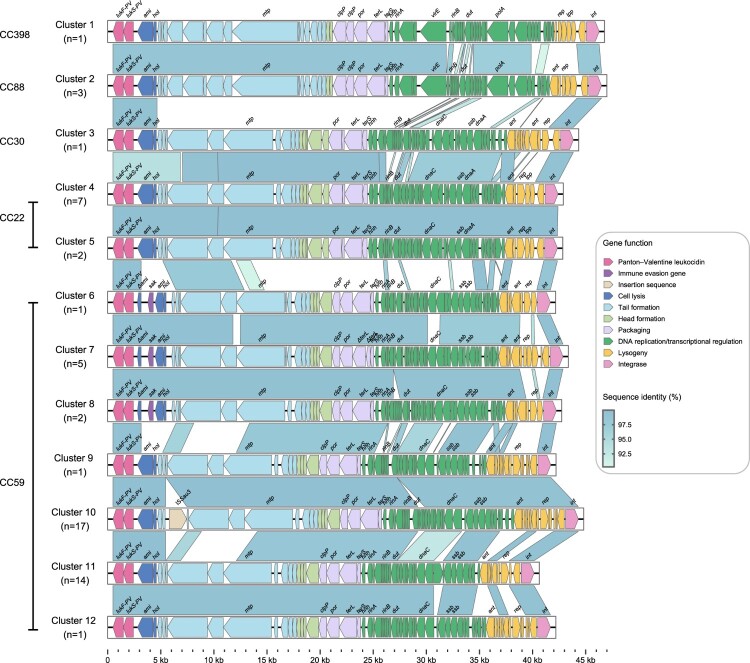
Linear comparison of PVL prophages among PVL-positive strains. Arrowed boxes represent genes that are coloured according to functional classification.

The *lukSF-PV* genes are located on a prophage and *in vitro* evidence suggests that these genes can be transferred from one strain to another via phage transduction [26]. Consequently, we compared the linear structures of PVL prophages among the PVL-positive strains. In this study, all MRSA strains carrying the PVL-encoding prophage belonged to icosahedral head group II. Cluster analysis based on the similarity of PVL-encoding prophage sequences categorized the 131 prophages into 12 sequence clusters (Clusters 1-–2), with Clusters 10 and 11 accounting for over 56.4% of all prophages and predominantly associated with ST59. Notably, we found that PVL and *sak* were co-located within the ΦSa2 prophages of Clusters 6, 7, and 8 of CC59. As shown in Figure 5, the PVL-encoding prophage sequences consisted of a PVL-encoding module, lysis module, structural module (packaging, head, and tail), DNA replication/transcriptional regulatory module, and lysogenisation module (including integrase). The sequence differences between the clusters were concentrated in the DNA replication/transcriptional regulatory, lysogenisation, and tail structural modules. The mosaic structure of the phage genome can be broadly categorized into two regions. First, the region common to all PVL phages encompasses five genes: *int, lukS, lukF, hol*, and *ami*. Second, the region responsible for encoding the structural module is essential for phage grouping. This region is unique to each PVL phage, although some homologous sections are present.

Different STs carry distinct PVL prophage sequence clusters. The CC398 strains belonged to Cluster 1, whereas the CC88 strains belonged to Cluster 2. The CC22 strains belonged to Clusters 4 (*n* = 7) and 5 (*n* = 2). The presence of various PVL-encoding prophages in different ST types indicates that each ST independently acquired PVL-encoding prophages. Notably, the major sequence clusters were highly similar across different CCs, suggesting that these clusters may have originated from a common ancestor and undergone recombination events during evolution.

Using the PVL allelic genes encoded by ΦPVL108 as reference sequences, we identified six unique SNPs among all PVL-positive MRSA strains in this study. Four SNPs were found in the *lukS-PV* gene, and two were found in *lukF-PV*. According to the classification scheme proposed by O’Hara et al. [27] and considering the SNP sites discovered in this study, 55 PVL allelic genes were classified into six haplotypes: H1a, H1d, H1e (a novel haplotype), H1f (a novel haplotype), H1 g (a novel haplotype), and R. H1d (*n* = 13) and H1a (*n* = 25) were the most common haplotypes (Figure S1). Notably, SNPs located in H1e, R, and H1 g could result in amino acid changes, including isoleucine replacing leucine at position 55 in *lukS-PV* (p.55I), arginine replacing histidine at position 176 in *lukS-PV* (p.176R), and tyrosine replacing histidine at position 231 in *lukS-PV* (p.231Y). Furthermore, the predominant haplotypes correlated with different CCs: strains encoding H1a primarily belong to CC59 and CC22, those encoding H1d were all CC59, and those encoding H2 were CC59 and CC88. This result suggests that the *pvl* genes carried by strains of various CCs have evolved independently.

## Discussion

Incidence data regarding SSTIs in the United States reveal a declining trend, while hospitalization rates are rising [28]. Although SSTIs are generally associated with low mortality, there is notable concern regarding the high rates of treatment failure and recurrent infections. Recent data from the United States published in 2013 reported an incidence of approximately 500 clinically diagnosed SSTIs episodes per 10,000 person-years [28, 29]. To our knowledge, this is the first study that compared the clinical characteristics of SSTIs caused by PVL-positive and PVL-negative MRSA isolates among inpatients in China and the genomic epidemiology of the isolated strains. The main findings were as follows: (i) PVL-positive SSTIs occurred more frequently in younger patients, frequently manifested as abscesses, and required incision and drainage instead of rigorous antimicrobial treatment; (ii) ST59 is currently the predominant clone in PVL-positive and PVL-negative SSTIs; and (iii) all sampled *pvl* genes seem to have a recent common ancestor and have disseminated through a combination of clonal expansion and horizontal transfer. Moreover, significant diversity was observed even within strains detected in the same facility.

PVL-positive MRSA-induced SSTIs are typically considered a therapeutic challenge with more severe/chronic clinical presentations, which impair the quality of life and mental health of patients. Our findings present an intriguing departure from this conventional understanding [30–32]. Contrary to expectations, we observed that patients with SSTIs caused by PVL-negative MRSA were older, had a higher prevalence of comorbidities, and had longer hospital stays than those with PVL-positive MRSA strains. One possible explanation may be the distinct baseline characteristics of the patient populations associated with PVL-negative and PVL-positive MRSA-induced SSTIs. Patients with PVL-negative MRSA-induced SSTIs may present with more complex medical conditions or exhibit greater susceptibility to complications, leading to a more conservative treatment approach and prolonged hospitalization. Moreover, most studies primarily focused on outpatient cases, whereas our study focused on hospitalized patients who often manifest more severe complications. Additionally, most patients with PVL-negative MRSA-induced SSTIs have secondary infections.

However, relying solely on screening patients for PVL carriage may not accurately assess disease risk. Our findings suggest that individual patient characteristics more significantly influence the manifestation and ultimate outcome of SSTIs than the PVL status of the MRSA strains. Despite the frequent association between *pvl* gene-carrying strains and invasive staphylococcal infections, emerging empirical data challenge the notion that PVL is the primary determinant of disease severity and prognosis. This assertion is supported by robust clinical investigations conducted in Australia, which systematically compared the outcomes between PVL-positive and PVL-negative strains across various invasive infections [33, 34]. These studies have consistently affirmed that the impact of PVL on outcomes is minimal, except for a notably increased utilization of surgical interventions in SSTI cases. Two distinct investigations that focused on clinical outcomes in individuals diagnosed with hospital-acquired pneumonia [35, 36] consistently demonstrated similar clinical progress and mortality rates across patients irrespective of PVL status. Importantly, these observations remained robust even after meticulous adjustments for potential confounding variables.

*In vitro* studies have encountered limitations in establishing a definitive association between the quantity of PVL toxins synthesized by diverse *S. aureus* strains and the intensity of clinical manifestations [37]. Furthermore, experimental research using animal models of SSTIs and pneumonia has failed to demonstrate a direct correlation between PVL status and clinical disease severity; the observed effect on disease development appears to be unrelated to specific bacterial strains [34, 38, 39]. Therefore, the importance of the PVL status in both basic research and clinical settings remains controversial.

A meta-analysis provided robust evidence showing the significant association between PVL and deep SSTIs [8], highlighting the tendency for infections to recur when not promptly diagnosed and treated [40]. One study reported an elevated risk of PVL carriage in patients with SSTIs [13]. Some studies have shown that community-acquired pneumonia (CAP) caused by PVL-positive MRSA strains frequently leads to adverse clinical outcomes, including extensive lung necrosis, multilobar infiltrates, leukopenia, hemoptysis, and severe sepsis [41, 42]. Although there is a clear association between CAP caused by PVL-positive MRSA strains and adverse patient outcomes, the role of PVL in the pathogenesis of pneumonia remains contentious [38, 43]. Contrary to previous concerns about the potential exacerbation of disease severity in PVL-positive cases, one study showed that, compared to HAP/VAP patients infected with PVL-negative MRSA, those infected with PVL-positive MRSA strains do not exhibit an increased risk of mortality [35]. These studies challenge the historical emphasis on PVL as a decisive virulence factor in MRSA infection [38, 41]. Although PVL remains an important aspect of MRSA epidemiology, especially in community settings, its impact on the severity of hospital-acquired infections, particularly HAP/VAP, may be less pronounced than previously assumed.

However, a substantial portion of the literature linking the *pvl* gene to invasive diseases originates from the United States, where the prevalent USA300 strain is resistant to methicillin in most community-acquired and some hospital-acquired cases [44, 45]. This is in stark contrast to reports from Europe, where a significant proportion of PVL-positive strains are methicillin-sensitive [46, 47]. In Australia, half of the community MRSA clones are PVL-positive, contributing significantly to the burden of diseases caused by PVL-positive MSSA strains [48]. The presence of USA300 is a potential confounding factor in the epidemiological association between invasive diseases and PVL status. As researchers explore the role of PVL in environments where USA300 is less prevalent, the potential impact of this confounding effect has become increasingly apparent.

Studies have also demonstrated that PVL-positive MRSA infections are more likely to be genuinely community-acquired, affecting individuals with no previous exposure to healthcare settings, in contrast to PVL-negative strains [47, 48]. However, 40% of PVL-positive strains were detected in hospital-acquired infections in the present study. Studies have reported an escalating incidence of healthcare-associated infections attributable to PVL-positive MRSA strains [49, 50]. Our study provides empirical support for the hypothesis that PVL-positive MRSA variants have transitioned into burgeoning nosocomial pathogens.

The observation that individuals with PVL-positive MRSA-induced SSTIs had a higher likelihood of requiring surgical intervention than those with PVL-negative infections underscores the importance of clinical considerations. This finding is consistent with previous research suggesting that PVL-positive MRSA strains tend to induce more severe localized diseases, often necessitating surgical intervention for proper management [8]. Furthermore, our study sheds light on the potential benefits of timely and appropriate surgical treatment, particularly focusing on proper abscess drainage in patients with PVL-positive SSTIs. The significant reduction in abscess resolution time associated with these surgical interventions underscores their effectiveness in expediting healing. This aligns with the principles of the surgical management of SSTIs, where incision and drainage are often crucial to alleviate pressure, remove infected material, and facilitate rapid recovery. It is important to note that while surgical intervention is beneficial, the choice of antibiotics remains crucial in the overall management of these infections. Combining appropriate antibiotics with surgical procedures can provide a comprehensive approach to treating PVL-negative SSTIs and improve both clinical outcomes and patient recovery. This holistic approach to patient care should be considered when managing individuals with PVL-negative SSTIs, especially in cases where timely intervention can significantly affect disease progression and resolution.

This is the first study to employ whole-genome sequencing technology to analyze the diversity of complete PVL prophage sequences across different CCs in China. Previous analyzes relied primarily on PCR schemes targeting specific regions of the prophage genome [51, 52], potentially missing unknown or newly identified PVL-encoding prophages. Furthermore, our analysis revealed that the major PVL-encoding prophage sequence clusters exhibited a high degree of similarity within the same CCs, implying that they shared a common ancestral origin and underwent recombination events that occurred during evolution. Notably, we found that PVL and *sak* were co-located within the ΦSa2 prophages within multiple CC59 clusters (Clusters 6, 7, and 8) and maintained for at least seven years. This is of particular interest because *sak* encodes a staphylokinase, which functions as an anti-opsonic immune evasion protein by transforming human plasminogen into active plasmin. Plasmin, deposited on the bacterial surface, is involved in the degradation of IgG and C3bf, thus helping bacteria inhibit phagocytosis by neutrophils [53]. Therefore, the co-occurrence of PVL and *sak* within the mobile ΦSa2 prophage is noteworthy, and whether this may contribute to the further prevalence of CC59 and whether there is a synergistic interaction between these two genes will be a focus of future research.

A previous investigation of sequence variations in the *pvl* genes and PVL phages among *S. aureus* strains collected from children in mainland China highlighted CC59 as the most prevalent PVL-positive MRSA clone [54]. Our previous surveillance study of MRSA in China indicated that CC59 is a prominent PVL-positive CC [55]. The current study expands on these findings by uncovering a substantial proportion of PVL-positive CC59 isolates and other PVL-positive CC59 clones associated with HA-MRSA infections. Collectively, these observations suggest the infiltration of PVL-positive ST59 clones into hospital settings, providing a potential explanation for the increased prevalence of PVL in HA-MRSA isolates. In addition, we observed an increased occurrence of SCC*mec* types IV and V in HA-MRSA isolates. Compared with SCC*mec* types II and III, MRSA-IV and MRSA-V strains exhibit smaller SCC*mec* elements [56]. Previous studies suggested that smaller SCC*mec* cassettes can alleviate bacterial fitness burdens [57]. Therefore, the increasing prevalence of CA-MRSA clones may contribute to the accommodation of smaller SCC*mec* cassettes carrying fewer resistance genes, thereby reducing their potential health burden. This indicates that SCC*mec* types IV and V, which are more sensitive to antimicrobials and exert less pressure, are gradually replacing larger multidrug-resistant SCC*mec* types, such as SCC*mec* II and III, in healthcare environments.

This study has several limitations. The chief among these is that the present findings emanate from a single medical institution and thus might not fully represent the broader Chinese population. While acknowledging the restricted scale of our study, it is noteworthy that patients with PVL-positive MRSA-induced SSTIs exhibited a higher propensity to experience severe localized pathologies, necessitating the implementation of incision and drainage procedures. It is also imperative to highlight that the decision to pursue incision and drainage as a treatment course was made before the molecular test results became accessible to medical practitioners. Despite the divergent outcomes observed in recent animal-based investigations comparing the virulence and fatality of PVL-positive and isogenic PVL-negative MRSA strains [34, 43], clinical studies have consistently established a link between the presence of *pvl* genes and heightened inflammatory reactivity, along with intensified local pathological manifestations [58]. Additional studies are required to evaluate the roles of other virulence genes in patient management and infection control. In addition, the use of draft MRSA genomes could potentially influence our analysis, owing to the presence of nucleotide gaps and errors. Moreover, the composition of MRSA bacterial populations within our collected samples showed the prevalence of the more sensitive ST59 lineage identified in cases of SSTIs. This diversity aligns with our previous study concerning the prevalence of clinical MRSA strains in China [55]. ST59, which is more susceptible to antimicrobials, has become the dominant MRSA clone in China.

## Declaration of competing interest

The authors declare no conflicts of interest. This work was supported by The National Key Research and Development Program of China (grant number 2021YFC2300300) and Research Project of Jinan Microecological Biomedicine Shandong Laboratory (JNL-2022006B).

## Supplementary Material

figure_S1

Supplementary_material_data_of_ARG_and_virulence

Supplementary_figure
