# Supplementary material for: Epidemiology and clinical features of Skin and Soft Tissue Infections Caused by PVL-Positive and PVL-Negative Methicillin-Resistant Staphylococcus aureus Isolates in inpatients in China: a single-center retrospective 7-year study
Source: Emerg Microbes Infect. 2024 Feb 7;13(1):2316809. doi: 10.1080/22221751.2024.2316809 (PMC10883109; doi:10.1080/22221751.2024.2316809)
Supplement: figure_S1 [file TEMI_A_2316809_SM1789.pdf]

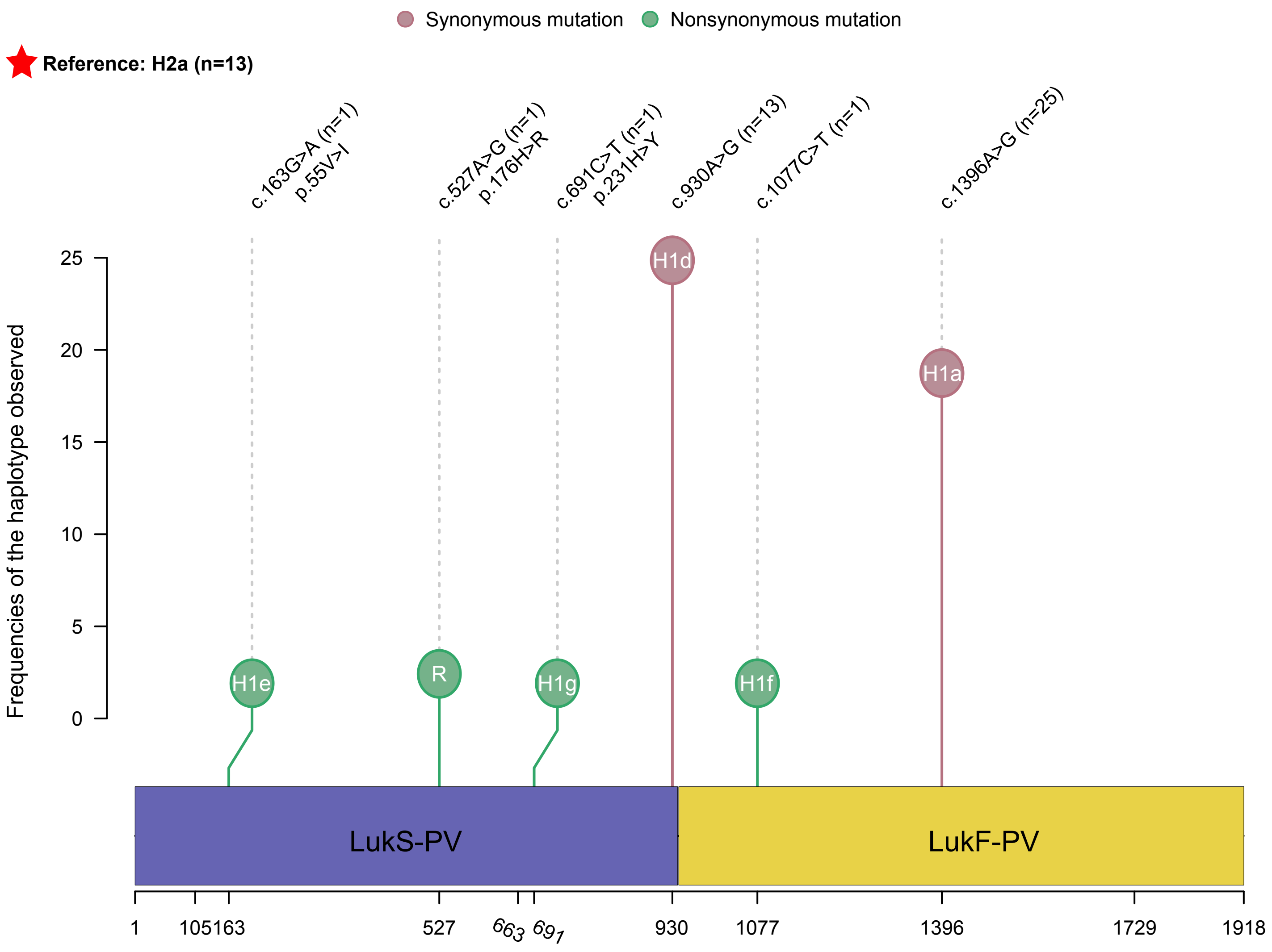

Synonymous mutation
Nonsynonymous mutation
Reference: H2a (n=13)
c.163G>A (n=1)
p.55V>I
c.527A>G (n=1)
p.176H>R
c.691C>T (n=1)
p.231H>Y
c.930A>G (n=13)
c.1077C>T (n=1)
c.1396A>G (n=25)
Frequencies of the haplotype observed
25
20
15
10
5
0
H1d
H1a
H1e
R
H1g
H1f
LukS-PV
LukF-PV
1
105
163
527
663
691
930
1077
1396
1729
1918
