# Supplementary material for: Epidemiology and clinical features of Skin and Soft Tissue Infections Caused by PVL-Positive and PVL-Negative Methicillin-Resistant Staphylococcus aureus Isolates in inpatients in China: a single-center retrospective 7-year study
Source: Emerg Microbes Infect. 2024 Feb 7;13(1):2316809. doi: 10.1080/22221751.2024.2316809 (PMC10883109; doi:10.1080/22221751.2024.2316809)
Supplement: Supplementary_figure [file TEMI_A_2316809_SM1780.docx]

**Figure S1. *LukSF-PV* sequence variants found in PVL+ methicillin-resistant *Staphylococcus aureus* (MRSA) isolates.**
